# Characteristics of commercial determinants of health research on corporate activities: A scoping review

**DOI:** 10.1371/journal.pone.0300699

**Published:** 2024-04-26

**Authors:** Raquel C. Burgess, Kate Nyhan, Naisha Dharia, Nicholas Freudenberg, Yusuf Ransome

**Affiliations:** 1 Department of Social and Behavioral Sciences, Yale School of Public Health, New Haven, Connecticut, United States of America; 2 Harvey Cushing/John Hay Whitney Medical Library, Yale University, New Haven, Connecticut, United States of America; 3 Department of Environmental Health Sciences, Yale School of Public Health, New Haven, Connecticut, United States of America; 4 Bachelor of Health Sciences Program, Faculty of Health Sciences, McMaster University, Hamilton, ON, Canada; 5 Department of Community Health and Social Sciences, CUNY Graduate School of Public Health & Health Policy, New York City, New York, United States of America; LSHTM: London School of Hygiene & Tropical Medicine, UNITED KINGDOM

## Abstract

**Introduction:**

Business practices have influenced human health for centuries, yet an overarching concept to study these activities across nations, time periods, and industries (called ‘the commercial determinants of health’ (CDH)) has emerged only recently. The purpose of this review was to assess the descriptive characteristics of CDH research and to identify remaining research gaps.

**Methods:**

We systematically searched four databases (Scopus, OVID Medline, Ovid Embase, and Ovid Global Health) on Sept 13, 2022 for literature using CDH terms that described corporate activities that have the potential to influence population health and/or health equity (n = 116). We evaluated the following characteristics of the literature: methods employed, industries studied, regions investigated, funders, reported conflicts of interest, and publication in open-access formats.

**Results:**

The characteristics of the articles included that many were conceptual (50/116 articles; 43%) or used qualitative methods (37; 32%). Only eight articles (7%) used quantitative or mixed methods. The articles most often discussed corporate activities in relation to the food and beverage (51/116; 44%), tobacco (20; 17%), and alcohol industries (19; 16%), with limited research on activities occurring in other industries. Most articles (42/58 articles reporting a regional focus; 72%) focused on corporate activities occurring in high-income regions of the world.

**Conclusions:**

Our findings indicate that literature that has used CDH terms and described corporate practices that influence human health has primarily focused on three major industries in higher-income regions of the world. Qualitative methods were the most common empirical method for investigating these activities. CDH-focused investigations of corporate practices conducted by less-studied industries (e.g., social media) and in lower-income regions are recommended. Longitudinal quantitative studies assessing the associations between corporate practices and a range of health outcomes is also a necessary next step for this field.

## Introduction

The activities of businesses have influenced human health for centuries. The mechanisms through which commercial entities can influence population health, both positively and negatively, are diverse. For example, commercial entities can negatively influence health by promoting the over-consumption of unhealthy products (e.g., tobacco products, alcohol, ultra-processed foods), with implications for the non-communicable disease crisis [1–3]. Companies can positively influence health by providing employees with adequate safety protections, which may reduce the rates of occupational injuries [4].

Though research on the health implications of business practices has occurred throughout the history of public health, it was only recently that an overarching concept was developed to guide the study of these influences across industries, nations, and time periods [5,6]. This concept is the commercial determinants of health (CDH), which has been defined as “the ways in which actors and structures operate to generate profit, and thereby influence patterns of health, disease, injury, disability, and death within and across populations” [5]. Similar to the concept of the social determinants of health (SDH), the CDH provides a lens to understand the conditions that shape people’s lives and their potential to attain optimal health [5,7].

Though terms related to the CDH (e.g., “industrial epidemics”) were used in the public health literature as early as 2005 [8], the specific terms “commercial determinants of health” and “corporate determinants of health” were used for the first time in 2013 [8], when Millar [9] described the positive and negative health impacts of corporations and West and Marteau [10] discussed the tension between profit incentives and public health. Since its inception, a relatively small group of dedicated scholars have worked to develop the concept and describe the structures driving the CDH (e.g., neoliberalism, capitalism, corporate rights), the activities through which the CDH manifest (e.g., lobbying, marketing), and the consequences of the CDH (e.g., non-communicable disease, premature death) [6,11]. In March of 2019, de Lacy-Vawdon and Livingstone conducted a systematic search of the CDH literature to understand how the term has been defined and evaluate the strengths and weaknesses of the literature base to date [6]. Based on the 33 articles meeting their criteria at that time, they concluded that research on the CDH was “relatively underdeveloped” and lacked specificity. They reached this conclusion in part because they found the literature to be primarily descriptive and conceptual, limited in its use of original data, and wanting of more systematic forms of investigation such as structured case study methodology [6].

Yet research on the CDH has proliferated in recent years [12], with at least 155 articles using CDH terms published between April 2019 and Sept 2022 (according to *The Lens* database [13]). In line with de Lacy-Vawdon and Livingstone’s call for increasing specificity, many have suggested that a research priority for the CDH field is to develop specific metrics that can be used to measure and track changes in corporate activities over time [5,12,14]. Our author team recently completed a scoping review of literature that used CDH terms and discussed corporate activities that can influence population health and health equity. We qualitatively synthesized the articles included in this review to develop a typology (called the Corporate Influences on Population Health (HEALTH-CORP) Typology) that describes a common set of corporate practices with the potential to influence population health that have been observed across industries [15, 16]. The intent is for this typology to serve as the basis for the development of specific metrics to measure these practices.

Importantly, the body of articles retrieved during this process provided us with the opportunity to assess the descriptive characteristics of this literature and identify remaining research gaps for the CDH field. Specifically, we sought to address the following primary research question: *How have researchers using the CDH concept studied corporate activities that can influence population health and health equity*? *Specifically*, *what methods have they used to study these activities*, *what industries have they investigated*, *and in what regions have these activities been studied*? Moreover, given the limited recognition and funding availability that CDH research faces [17,18], we also sought to address the following secondary question: *What are the operational characteristics of this literature such as the funding bodies supporting it*, *the conflicts of interest (COIs) reported by its authors*, *the journals in which it is published*, *and its accessibility (open-access status)*?

In the following sections, we report the methodology we employed to study these questions, describe our findings, and propose three major research priorities for the CDH field that were informed by our findings.

## Methods

We conducted a scoping review of articles that used CDH terms and described corporate activities that have the potential to influence population health and health equity. We have reported our methods and findings in accordance with the PRISMA extension for scoping reviews (PRISMA-ScR) [19]. No protocol was published.

### Search strategy

Our search strategy was based on the strategy employed de Lacy-Vawdon and Livingstone [6], which we then broadened in consultation with a specialist public health librarian (K.N.). Specifically, in addition to conducting title, abstract, and keyword searches (T-Ab-Key) using CDH terms (e.g., “commercial determinants of health”, “corporate determinants of health”), we also performed T-Ab-Key searches for related terms (e.g., “corporate political activity”). During the full-text screening phase, we applied exclusion criteria based on the presence or absence of CDH terms (i.e., “commercial determinant(s)”, “corporate determinant(s)”) in the full-text of the articles. We employed this strategy to maximize the number of articles we captured that are relevant to our aim, as it allowed us to capture articles that used CDH terms in the text of the article but not in the T-Ab-Key.

We searched Scopus, OVID Medline, Ovid Embase, and Ovid Global Health with no date restrictions. The search was conducted on Jan 4, 2022 and the same search was conducted again on Sept 13, 2022 to identify newer articles published since the previous search. The full search string for each database is presented in the Supporting Information, Appendix 1 in S1 File.

### Eligibility criteria

Eligible articles were those written in the English language that described or investigated activities (i.e., decisions, strategies, or other actions) that corporations or those acting on behalf of corporations engage in that have been demonstrated to or have the potential to influence population health and/or health equity. No restrictions were placed on the publication date, the date the data was collected, the methods employed, or the industry under study. All types of published articles were included, including non-empirical articles (i.e., commentaries, conceptual articles, book chapters). Articles were also included if corporate activities were not the main focus of the article but the article nevertheless contained discussion about corporate activities in relation to population health and health equity.

Articles were excluded if they: a) did not mention “commercial determinant(s)” or “corporate determinant(s)” within the T-Ab-Key or full-text of the article (or if the respective terms only appeared in the article’s reference section), b) did not describe activities, decisions, or strategies that were made by corporations or on behalf of corporations that were demonstrated to or had the potential to influence population health, or c) the full-text of the article was not available in English. Moreover, full-length books were excluded for feasibility purposes.

### Screening

Duplicates were removed before and during title and abstract screening using Mendeley [20] and Rayyan software [21] and manual de-duplication. The titles and abstracts were screened in Rayyan [21], a web service for organizing systematic reviews, by one author (R.B.). Eligible full texts were then retrieved and screened by the same author. Following full-text retrieval, articles that did not mention CDH terms in the T-Ab-Key or full-text of the article were excluded, a process conducted using EndNote’s [22] ‘Smart Group’ feature.

### Data extraction

Data was charted by the first (R.B.) and third author (N.D.). Charting fields included the methods employed, industries discussed, regions studied, reported funding, reported COIs, journal in which the article was published, and open-access status (retrieved via *The Lens* scholarly database open-access classifications). Charting fields were populated by one author and we did not employ double, independent charting for verification purposes. The rules we used to guide the classifications are provided in the Supporting Information, Appendix 2 in S1 File.

### Data synthesis and analysis

Data was described narratively in the text and analyzed using descriptive statistics. Graphs depicting results were generated using Excel software [23] and the map of regions investigated was generated using the *choroplethr* package [24] in RStudio [25].

### Critical appraisal

Critical appraisal of the included articles, which is not generally performed for scoping reviews, was not conducted as part of this review [26].

## Results

### Article screening

**Fig 1:**
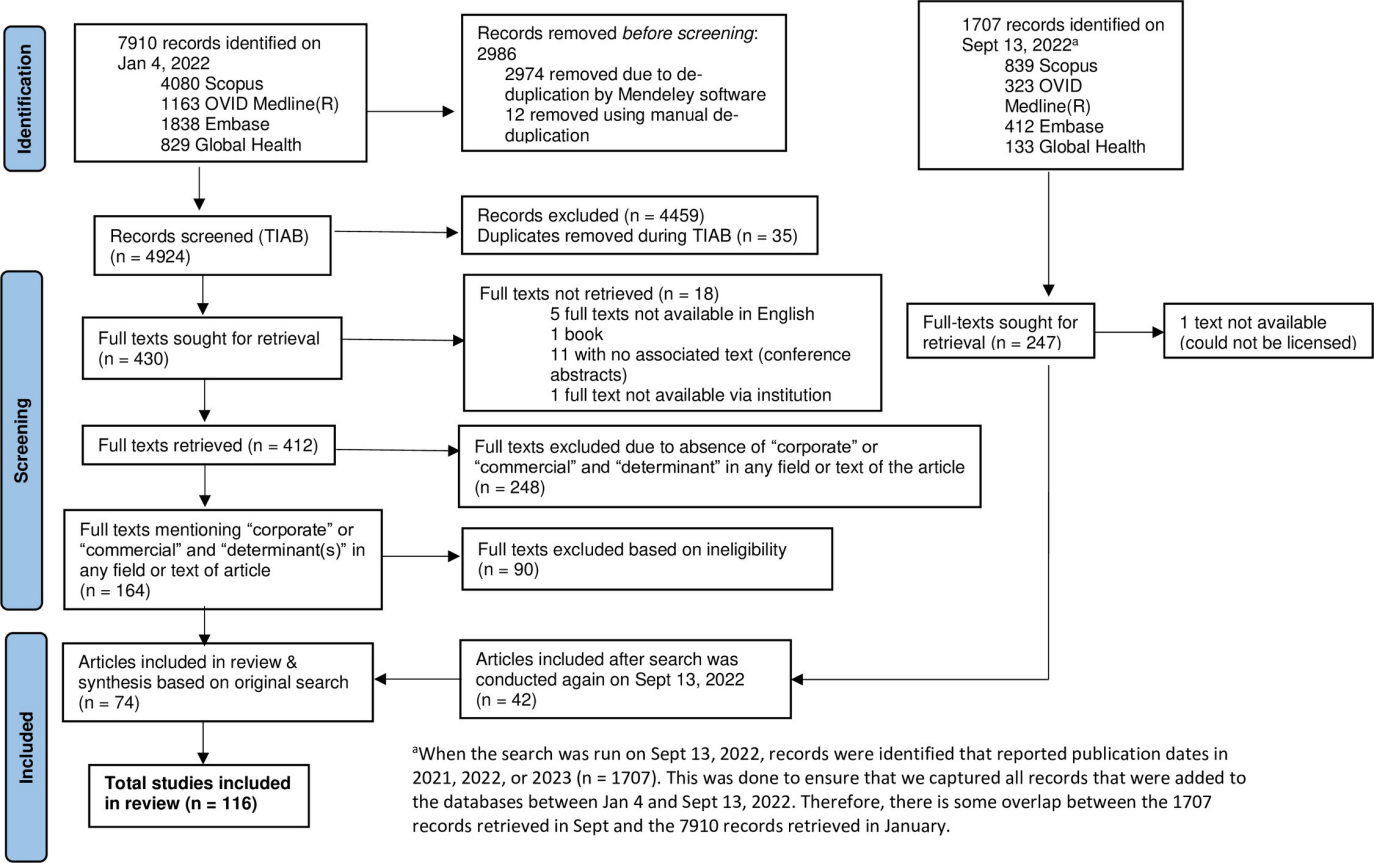
PRISMA flow diagram [27].

When the search was first conducted in January 2022, 7910 records were retrieved. After de-duplication, 4924 records were screened by title and abstract, after which 430 records remained. Full-texts could be found for 412 of these records. Of the 164 articles that used CDH terms in the full-text, 74 met the inclusion criteria. Another 42 articles met the inclusion criteria when the search was rerun in September 2022, resulting in 116 included articles (Fig 1).

### Characteristics of included articles

Below, we synthesize the descriptive characteristics of the included articles. Specifically, we describe the years in which the articles were published, the methods employed, industries investigated, regions studied, reported funders, reported COIs, journals in which the articles were published, and open-access status of the articles. Detailed information about the included articles is provided in the Supporting Information, Appendix 3 in S1 File.

#### Publication years

**Fig 2:**
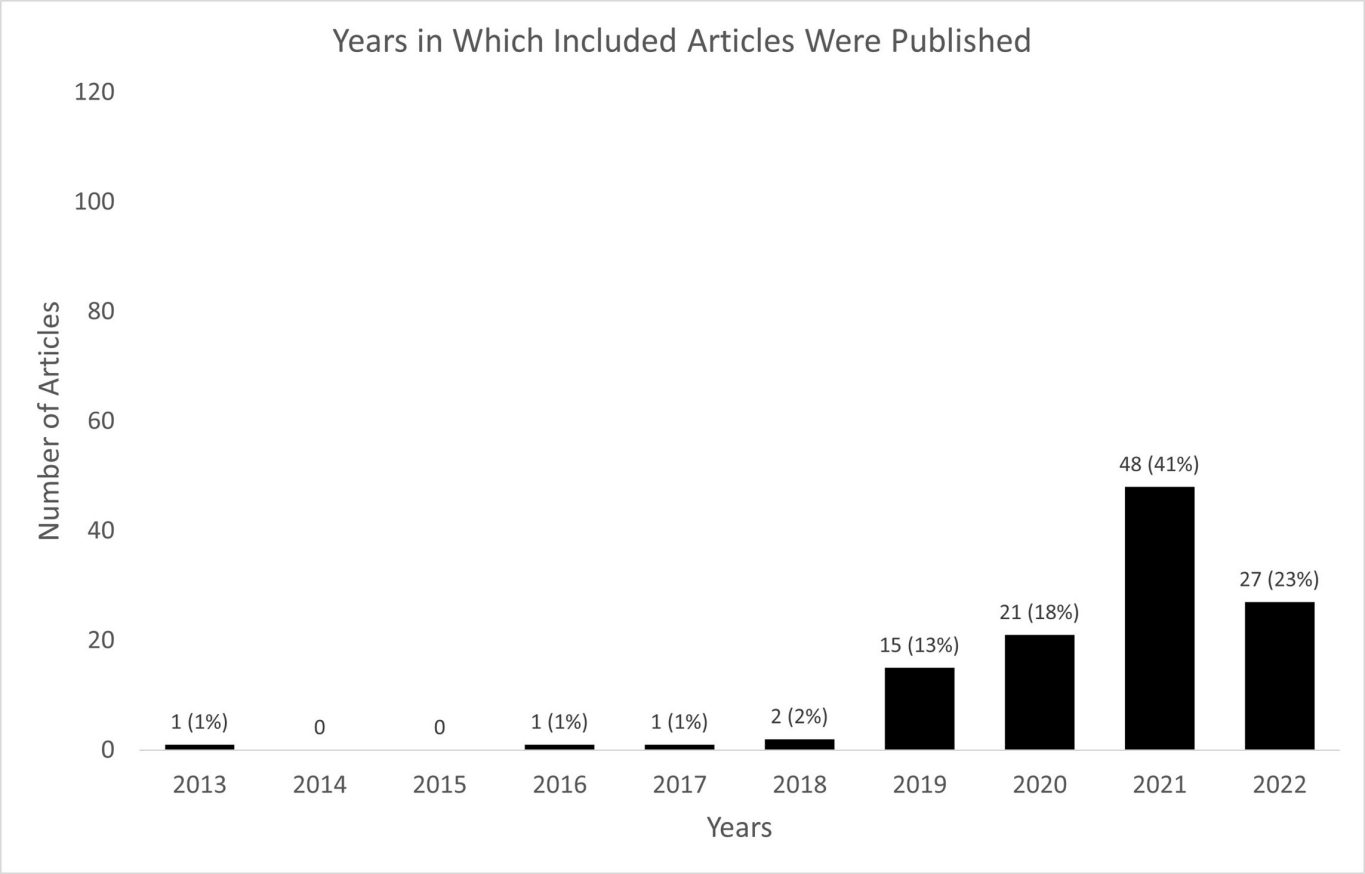
Years in which the included articles were published. Note that the numbers for 2022 are articles found as of September 13, 2022.

The earliest eligible article was published in 2013. Only a few (5; 4%) of the included articles were published between 2013 and 2018. Fifteen (13%) and twenty-one (18%) of the included articles were published in 2019 and 2020, respectively. A large proportion of the included articles were published in 2021 (48; 41%) and 27 (23%) were published in 2022 (as of the Sept 13^th^ search date) (Fig 2).

#### Industries

**Fig 3:**
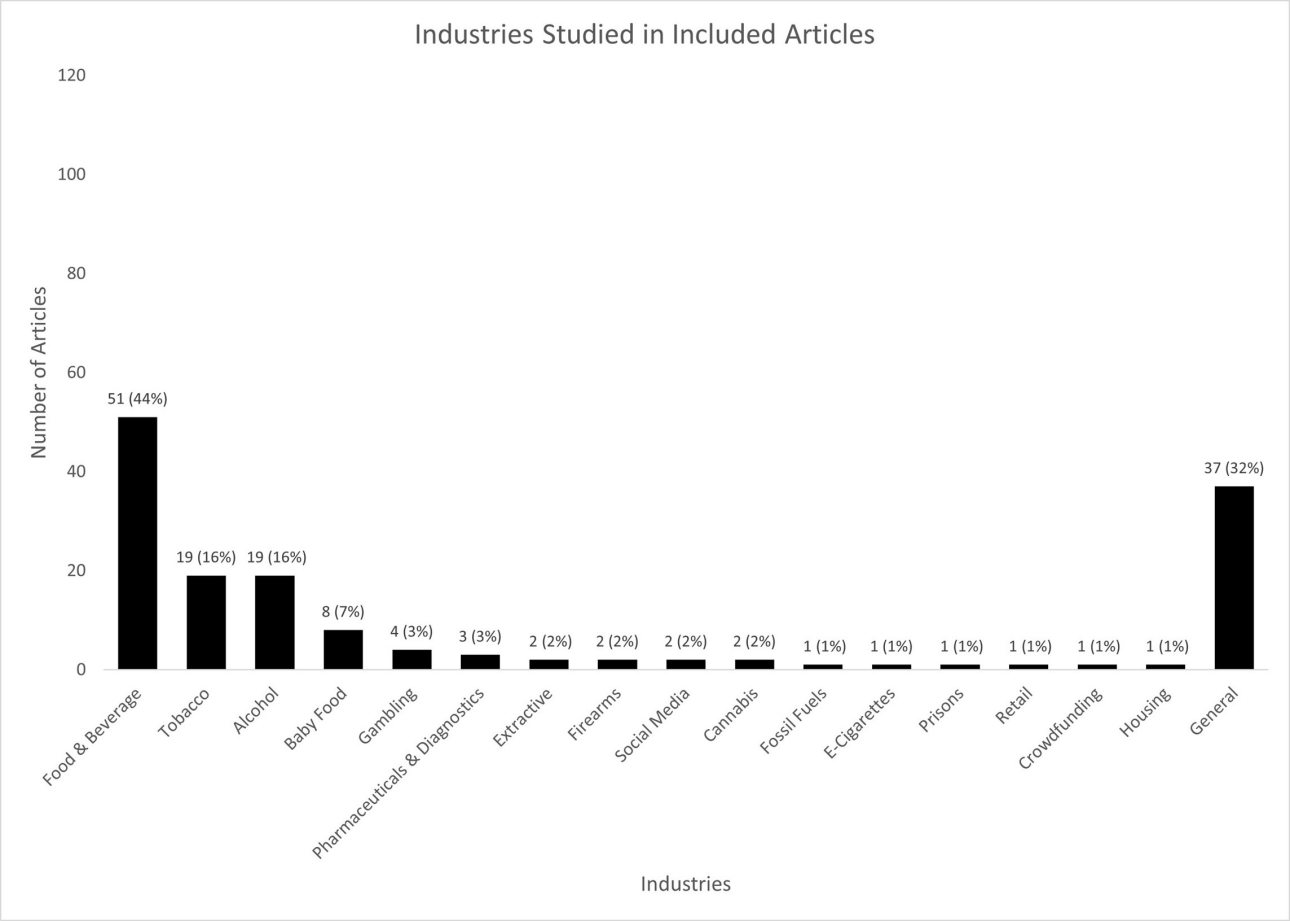
Industries studied in included articles. Articles were classified into a particular industry if they dedicated a substantial portion of the article to discussing that industry. Articles were classified as ‘general’ if they reported on corporate activities more broadly, in some cases using certain industries as illustrative examples. Articles could be classified into multiple industries; therefore, the total number of articles do not sum to 116 and the percentages do not sum to 100% (Supporting Information, Appendix 2 in S1 File).

The articles discussed corporate activities occurring in 16 different industries (Fig 3). The most frequently studied industries were food and beverage (F&B) (51 articles), tobacco (including e-cigarettes) (20), and alcohol (19) (67 unique articles, 58%). Other industries, some of whose products are emerging as potential health threats (e.g., social media, cannabis, gambling, e-cigarettes), were only discussed within a few of the included articles.

#### Regions

**Fig 4:**
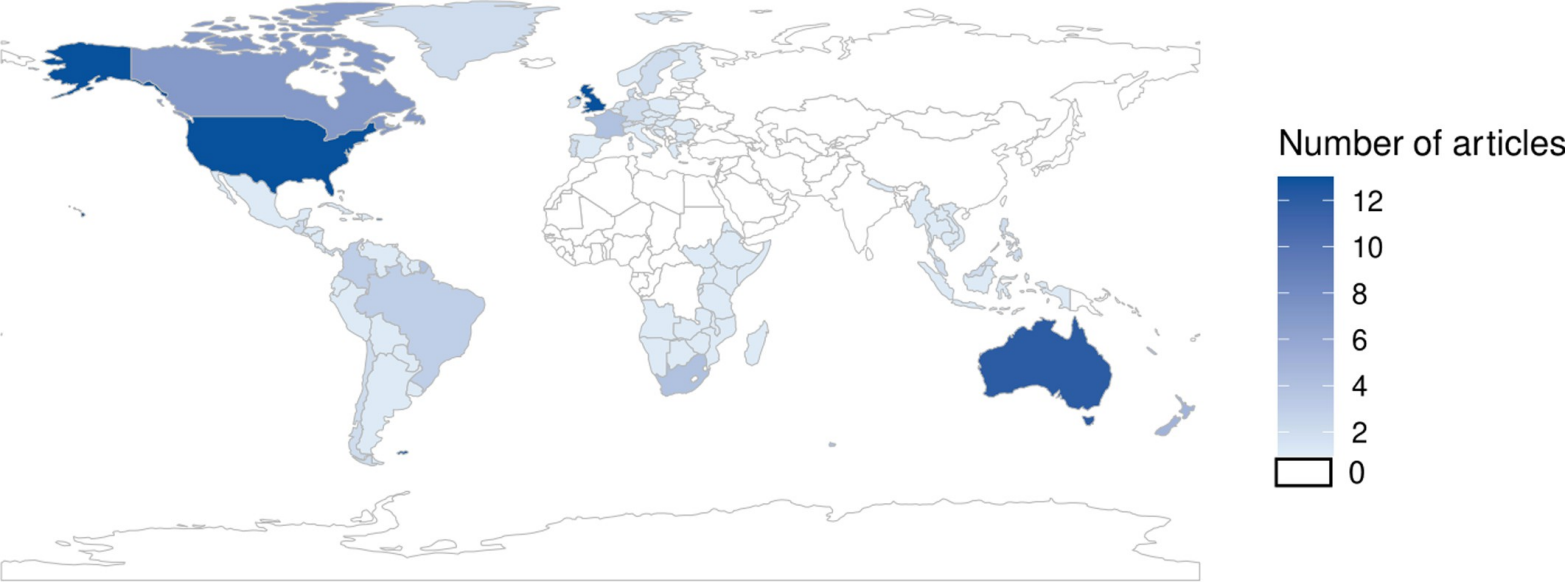
Regions that were the subjects of the included articles. Articles were classified to a particular region if they studied corporate activities occurring in that region, collected data from sources or participants from that region, or otherwise focused their article on that region. See Supporting Information, Appendix 2 in S1 File for more details on classification.

Thirty-four articles (34/116; 29%) did not discuss a particular region, and 25 (25/116; 22%) specifically reported a global scope. Of the articles reporting a specific regional focus (58 articles; 50%), most focused on high-income regions (42/58; 72%) (as defined by the World Bank classifications), including the United Kingdom (13 articles), the United States (12), and Australia (12). Another five articles (5/58; 9%) focused on a combination of high and middle-income countries and only nine articles (9/58; 16%) focused on middle-income countries, including South Africa (3 articles), Brazil (2) Columbia (2), and the Philippines (1). Two articles included a focus on low-income countries, one which discussed occupational health in East and Southern Africa and another which investigated the transfer of wealth from low-income countries to high-income countries (HICs) (Fig 4).

#### Corporate activities

The corporate activities described in the included articles reflected a range of activities including precarious employment practices, involvement of corporations in health education delivered in schools, tax avoidance, and lobbying against proposed health policies. In our complementary article, we describe these activities in detail. Specifically, our complementary article [15] describes how we engaged in a separate qualitative synthesis of the same body of articles included in this review for the purpose of developing a typology (i.e., the HEALTH-CORP typology) that describes the domains of influence (e.g., political practices, employment practices) through which commercial entities can influence population health. Within each identified domain, we categorized a set of specific corporate activities through which corporate influence on health is exerted. For example, the political practices domain includes the activity of political financing (associated with lower likelihood of health policy implementation [28]) and the employment practices domain includes the length of paid parental leave offered by the company (associated with maternal and child health [29]). The HEALTH-CORP typology is intended to facilitate a holistic understanding of the diverse mechanisms through which corporations can influence health and provides the groundwork for future efforts to measure these activities.

#### Methods employed in included articles

**Fig 5:**
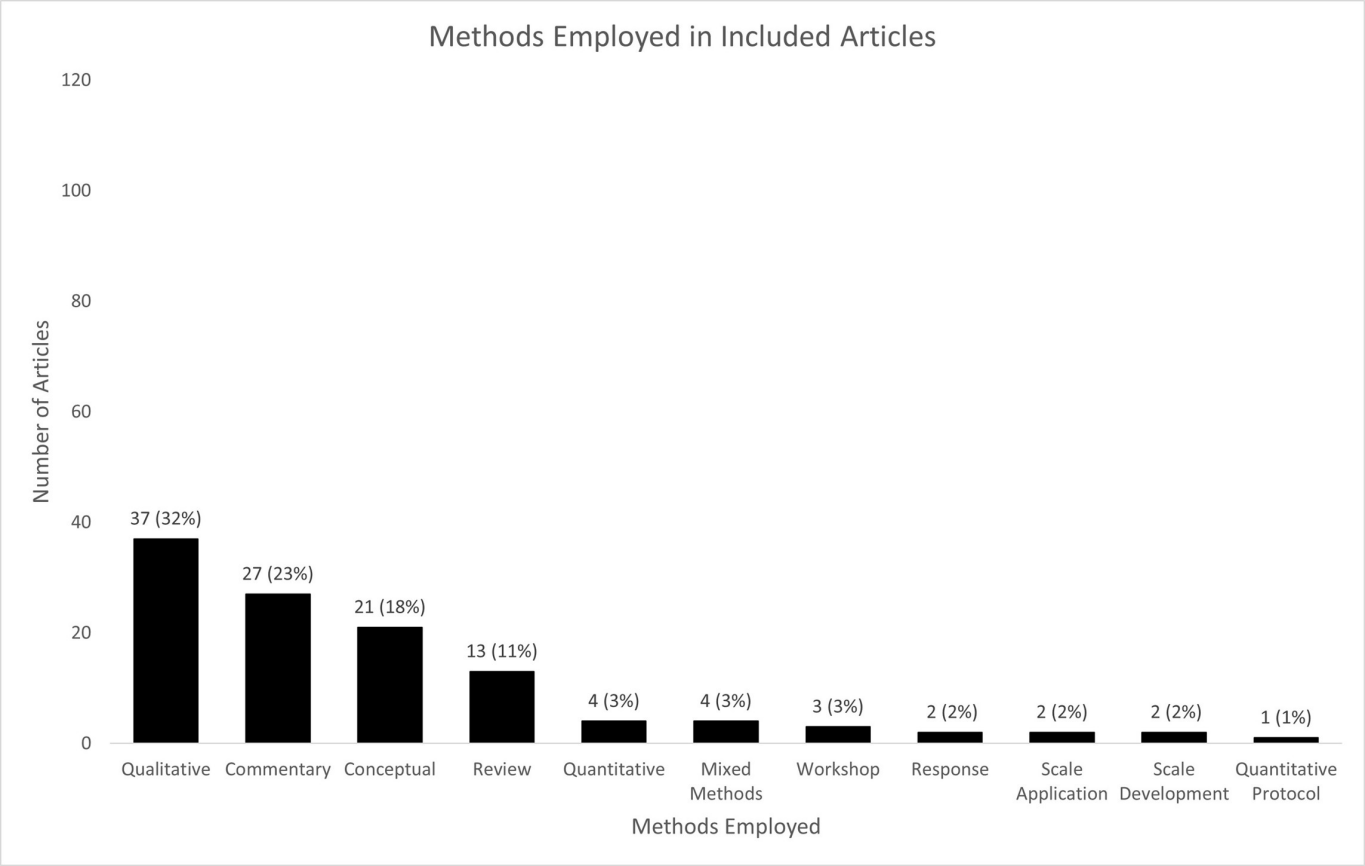
Methods employed in included articles. Articles were classified into the methods category deemed most applicable and were not classified into multiple categories. See Supporting Information, Appendix 2 in S1 File for more details.

Fifty articles (43%) were classified as conceptual articles, commentaries, or responses (Fig 5). These articles engaged in non-empirical processes such as describing corporate activities generally, proposing frameworks, or applying other concepts or previously-developed frameworks to reveal new perspectives on the CDH. For example, Liber [30] described how the regulatory stance framework could be used to consider the expansion of markets for products that are beneficial to health (e.g., vaccines) while creating conditional markets for products (e.g., e-cigarettes) that are harmful to some (e.g., youth) but may be beneficial to others (e.g., current smokers that switch to e-cigarettes). Kenworthy [31] used a CDH lens to describe the ways that for-profit crowdfunding platforms have negative impacts on global health and related concerns (e.g., by generating the conditions for the exposure of personal health data).

The empirical articles most commonly reported using qualitative methods (37/116 articles; 32%). Studies reporting qualitative methods were published starting in 2019 and frequently focused on the F&B industry (24/37, 65%). The aims of these studies generally related to identifying and documenting relevant industry strategies, such as strategies to influence the development or implementation of a proposed policy or industry attempts to influence the public’s knowledge about products and their health-related harms.

To achieve these aims, most articles reported using qualitative techniques (e.g., interpretive analysis, content analysis) to analyze industry-relevant documents (24/37, 65%) such as industry submissions to proposed health policies (e.g., the beverage industry’s response to South Africa’s proposed sugar-sweetened beverages tax[32]), social media posts (e.g., posts by the F&B industry related to the COVID-19 pandemic [33]), public-facing industry documents (e.g., resources from meat industry representatives on the environmental and health harms of meat [34]), and emails from industry actors (e.g., emails between employees of The Coca Cola Company and employees of the Centers for Disease Control and Prevention [35]) which were obtained via Freedom of Information requests. Other articles (4/38, 11%) reported conducting interviews with relevant stakeholders, such as former industry employees, consumers, policy actors, and members of civil society, to glean insights into corporate strategies. Nine articles (24%) reported using a combination of document and interview data. For example, Kroker-Lobos and colleagues [36] investigated the political practices of the F&B industry in response to two public health nutrition policies in Panama and Guatemala by collecting information from a combination of documents (e.g., F&B industry websites, conference websites). Given limited data availability in Central America, they triangulated and extended their findings by conducting interviews with stakeholders who had interacted with the industry during the policy making process.

To inform their study and guide their analysis, 27 (73%) of the articles reporting qualitative methods employed a pre-existing or author-derived theoretical (e.g., Conflict Theory [37,38]) or analytical framework (e.g., Thaler & Sunstein’s ten types of nudges [39,40]). The most commonly reported frameworks employed were Mialon and colleagues’ framework to monitor the corporate political activity of the food industry [41] (5/37, 14%) and the policy dystopia model [42] and its adaptation to the food industry [43] (the latter is an updated version of Mialon and colleagues’ CPA framework) (7/37, 19%). The findings from these studies often revealed the diverse, yet oft-repeated mechanisms through which corporation’s advance their interests in ways that can harm population health (e.g., use of messaging to normalize harmful commodity use [44,45]).

Only four articles used quantitative methods (4/116; 3%) and another article proposed a protocol for a quantitative study (1/116; 1%). These studies were diverse in scope, with some (2/5, 40%) employing descriptive statistics (e.g., to examine the distributions of wealth and income between firms and governments and shareholders [46]) and others employing (or proposing the use of) analytical techniques (e.g., to examine the effect of industry-sponsored messaging on risk perceptions [47]) (3/5, 60%). The most extensive analytical investigation was the construction and application of the Corporate Financial Index (CFI) by Allen, Wigley, and Holmer [28]. The CFI is comprised of six metrics (e.g., disclosure requirements for campaign donations) and seeks to quantify the extent to which corporations can influence health policy in countries via their finances. Using multivariate regression techniques and controlling for literature-derived confounders, the authors found that higher scores on the CFI were associated with a lower likelihood of implementation of WHO-backed commercial policies (e.g., tobacco taxation) using data from 172 countries.

Four articles described mixed methods approaches (3/116; 3%). These studies used some form of qualitative categorization and then quantified these findings [48,49]. For example, Du-Pont Reyes and colleagues [48] coded advertisements appearing on American English- and Spanish-language television on various characteristics and found that Spanish-language television contained higher rates of advertisements for alcohol and unhealthy F&Bs and lower rates of advertisements for healthy F&Bs. The methods employed by the remaining 20 articles are presented in Fig 3.

### Reported funders, reported conflicts of interest, journals, and open access

#### Status

**Fig 6:**
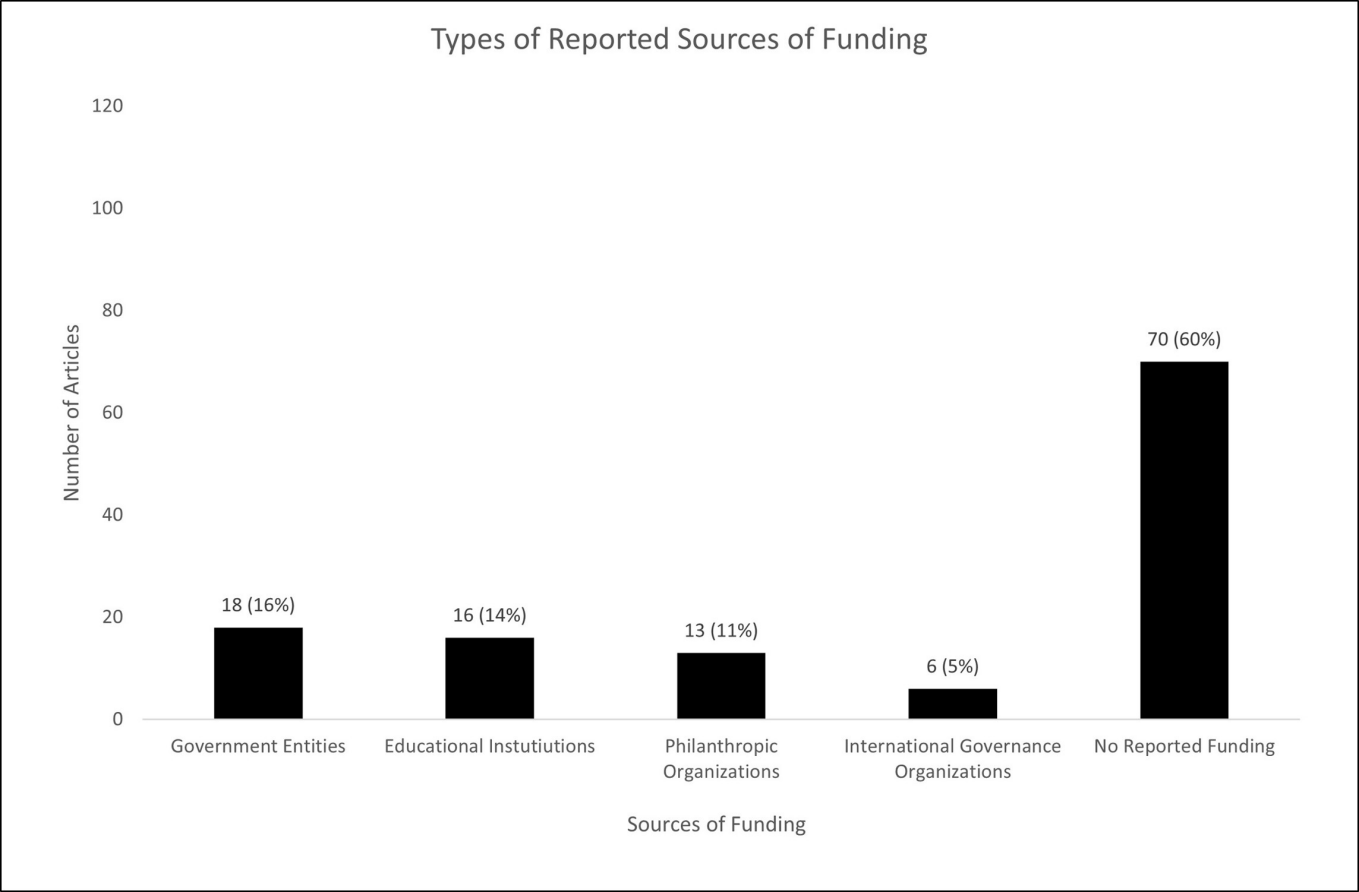
Types of funders providing research-specific funding reported in the included articles. Number of articles do not sum to 116 and percentages do not sum to 100 as some articles reported multiple funders of different types. See Supporting Information, Appendix 2 in S1 File for details on classification.

Less than half (46/116 articles; 40%) of the articles reported funding that was specific to the research described in the respective article. Reported sources of funding were government entities (18/46; 39%) (e.g., UK Medical Research Council), educational institutions (16/46 funded articles; 35%) (e.g., University of British Columbia), philanthropic organizations (13/46; 28%) (e.g., U.S. Right to Know; the Laura and John Arnold Foundation) and international governance organizations (6/46; 13%) (i.e., the World Health Organization) (Fig 6).

Twenty articles (17%) reported a COI related to the research. These ranged from relatively minor (e.g., an author is part of another research project funded by a government agency that levies money from alcohol sales [50]) to more severe (e.g., current and past funding from F&B industry actors (Nestlé, Danone (Bonafont) [51]).

**Fig 7:**
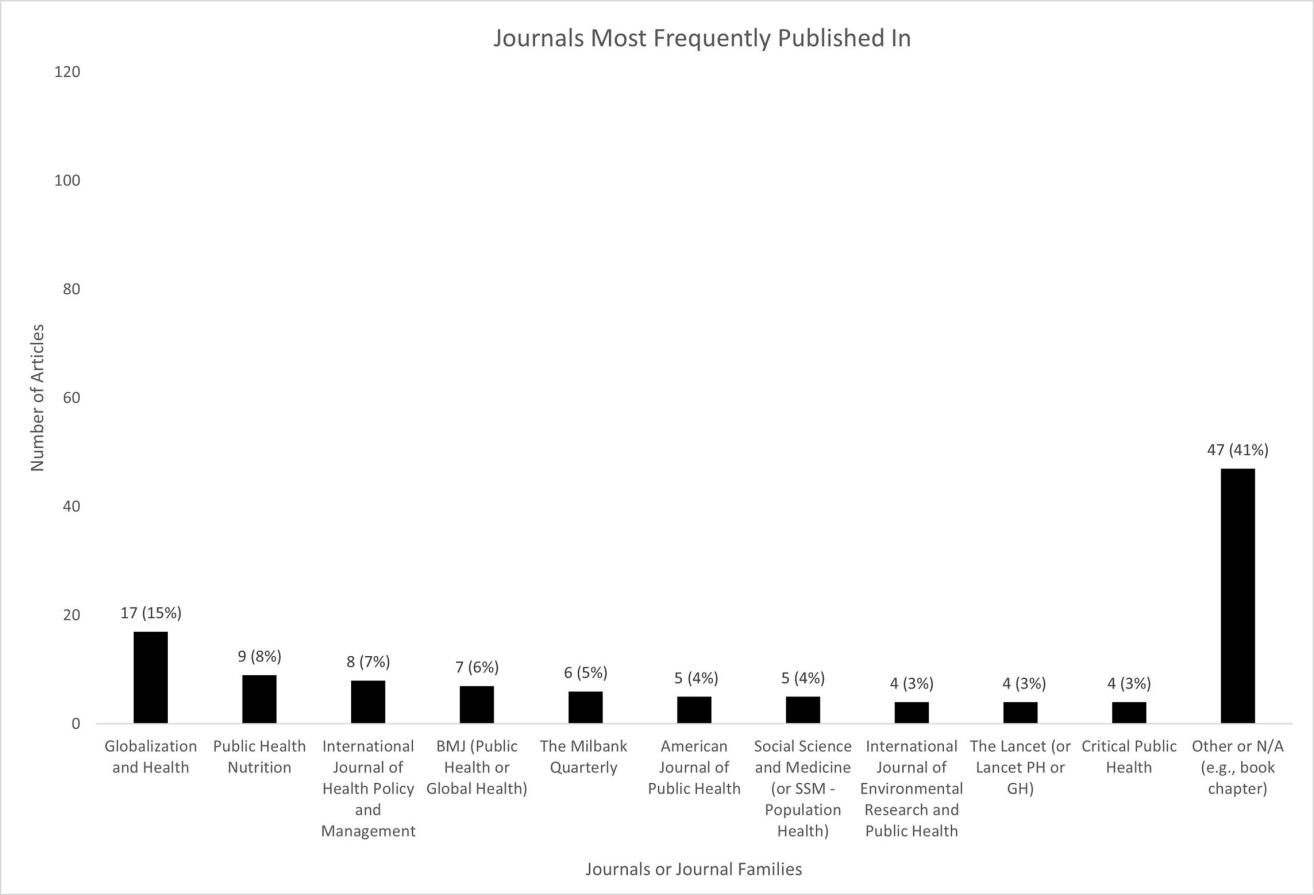
Journals in which the included articles were most frequently published.

Most of the research (101 articles; 87%) was published in open-access formats, such as via Creative Commons licenses. Sixty-nine articles (59%) were published in ten journals or journal families (presented in Fig 7).

## Discussion

In this article, we assessed the characteristics of literature (published prior to Sept 13, 2022) that used the CDH concept (and employed CDH terms) to describe corporate activities that can influence population health and health equity. The prevailing characteristics of these articles are that they focus on corporate activities conducted by the F&B, alcohol, and tobacco industries in higher-income regions of the world. These activities were primarily investigated using qualitative methods or discussed conceptually. Most of the research was not funded; those with funding received it primarily from educational institutions, government entities, or philanthropic organizations.

The findings from this study demonstrate that the use of the CDH concept to investigate corporate activities has increased over time in volume and specificity, with more recent articles employing qualitative methods to investigate specific corporate activities occurring in specific industries. The articles included in this review differ from those included in de Lacy-Vawdon and Livingstone’s review [6] because our review included CDH literature that specifically described corporate activities whereas de Lacy-Vawdon and Livingstone’s review employed broader eligibility criteria. Notwithstanding these differences, our findings suggest an improvement from the “primarily conceptual and descriptive” literature base that de Lacy-Vawdon and Livingstone found in 2019 [6] (see Supporting Information, Appendix 4 in S1 File for the overlap between articles included in both reviews).

Despite this progress, we found limited quantitative research on this topic. This gap is likely due to the challenges of examining statistical associations between activities that indirectly influence health, such as lobbying against health policies, to changes in specific health outcomes. Another barrier is the lack of available quantitative data, as a significant portion of data on corporate practices is proprietary and not publicly-available [52]. Despite these challenges, the quantitative studies included in this review indicate that it is possible to conduct quantitative research that supports previous inferences about the impact of specific corporate activities. For example, Maani and colleagues [53] generated their own data by conducting randomized trials of the impact of exposure to industry-sponsored messaging versus independent information about product-related harm on respondents’ certainty about product-related risk. This data provides empirical support for concerns raised about industry-sponsored messaging in previous literature [53]. Future work could focus on obtaining relevant data through, for example, corporate Environmental Social Governance (ESG) reports (published annually) and data aggregated by organizations such as the World Benchmarking Alliance [54], to investigate the associations between corporate activities and changes in health outcomes over time. This type of research could strengthen the case for addressing activities that perpetuate the CDH and provide easily digestible information on harms to decision-makers [5].

Our findings demonstrate that most of the CDH literature describing corporate activities has focused on the alcohol, tobacco, and F&B industries, a focus warranted by the staggering health burden of these industries’ products [55,56]. However, the application of the CDH concept to contemporary industries (e.g., social media, cannabis, e-cigarettes) that was seen in some the included articles is valuable as it strengthens the usefulness of the CDH concept as a tool for describing corporate activities and structures that influence health across industries. Further investigation of these industries may improve our understanding of how the CDH are operationalized similarly and differently within and across industries [57,58]. Moreover, a CDH perspective may reveal new understandings that will help us to address these industries’ health harms. For example, McCarthy and colleagues [59] illuminated the commercial determinants of gambling amongst older women, such as the addictive design of gambling machines, whereas previous research had focused primarily on individual and socio-cultural influences (e.g., gambling as a means to overcome loneliness).

Our findings suggest that most of the regionally-specific CDH research has focused on corporate activities occurring in higher-income regions of the world. This research gap could exist because a disproportionate number of CDH researchers may be based in HICs. Another reason may be that CDH researchers based in low- and middle-income countries (LMICS) may face greater challenges to publication (e.g., lack of available funding) [60]. Finally, corporate activities may be more difficult to study in contexts that lack relevant data sources such as national lobbying registries [61]. Likely, there are multiple, additive reasons for a lack of CDH research on LMICs.

Whatever the cause(s) may be, the lack of CDH research in LMICs is an important research gap for two reasons. First, given the different regulatory, cultural, and economic contexts of LMICs as compared to HICs, corporate activities in these regions may be distinct [62]. Second, due to the features of the current global economic system, LMICs are likely to be most affected by the CDH. For instance, LMICs often supply labour used to manufacture products but also have weaker regulations to protect workers [63,64]. Moreover, harmful industries (e.g., alcohol) have engaged in concentrated efforts to market their products in these ‘emerging markets’, where there are weaker regulations to prevent harm and lower-quality health systems to address the consequences [65–67]. For these reasons, the CDH field must find ways to address this gap. Ideally, this would involve determining the most pertinent reasons for a lack of LMIC-based research (for e.g., by interviewing LMIC CDH-based researchers) and then employing solutions designed to address these issues. For example, if the lack of CDH research on LMICs primarily stems from a disproportionate number of CDH researchers being HIC-based, efforts to partner with and build capacity with LMIC institutions may help to address this issue.

Finally, we found that less than half of the research in this review received specific funding for the research in question. The lack of funding could be limiting the field’s ability to conduct large scale studies, such as those evaluating the associations between corporate activities and specific health outcomes, that may require access to expensive datasets or large groups of participants. Funding for this type of research has the potential to improve as CDH work is further recognized and promoted by important institutions, such as the recent *Lancet* series [68] on the CDH and the upcoming report expected from the *World Health Organization*. Given the inherent conflict in accepting research funding from industry for CDH-related research, governments, universities, and appropriate non-profit funders (i.e., not industry front groups) will play an important role in supporting this research. However, avoiding the acceptance of funding connected to industry may be more difficult than it seems due to undisclosed or hidden relationships between non-profit organizations and corporations [69,70]. These obscured relationships may also mean that our ability to detect COIs by examining self-declared COIs that appear in journal articles is limited. Given the wealth of evidence linking industry research funding to bias across the research cycle [71], efforts to keep CDH research free of industry influence will be important to maintaining the integrity of our investigations.

Despite the lack of reported research funding, a substantial number of articles in this review were published in open-access formats, a promising step towards promoting and advancing the CDH concept, both from an academic and applied perspective. Though the CDH field may benefit from dedicated avenues (e.g., CDH-specific journals, conferences, or research centers) through which CDH research can be generated and disseminated, the drawbacks of this approach include the potential to further isolate the CDH field from more mainstream public health paradigms (e.g., the social determinants of health) [18].

### Strengths and limitations

The strengths of this review include that we were able to summarize the characteristics of a body of literature that spans multiple industries, disciplines, regions, and a relatively long time period using rigorous and transparent methods.

One of the most important limitations of our review is that we limited our eligibility criteria to articles that directly engaged with CDH terms, a decision made for feasibility purposes. As such, our review does not capture all articles in which authors have used the CDH *concept* (i.e., the idea that commercial practices are determinants of health and disease) to investigate corporate activities; it captures the subset that directly engaged with CDH *terms*. Therefore, our findings should not be interpreted to encompass the entire universe of research that has investigated corporate activities from a CDH perspective.

Another limitation results from our exclusion of non-English language and grey literature for feasibility purposes. Our lack of non-English language literature may account in part for the lack of literature found on corporate activities in Asia, Latin America, and parts of Africa. We may have also had better success in finding eligible literature on low-income regions by searching databases with better coverage of journals based in LMICs, such as *OpenAlex* [72]. However, given that a significant portion of transnational corporations are headquartered in English-speaking regions but conduct their activities internationally [73], we should expect to see even English-centric CDH research reporting on corporate activities occurring in LMICs.

Finally, all screening was conducted by the first author and was not verified by a second, independent screener. Similarly, some of the included articles did not fit neatly into one methods category. Though we applied rules to guide the methods classifications, the first author made the classification decision and these decisions were not verified by a second author.

## Recommendations for research priorities

Based on the findings from this scoping review, we recommend three research priorities for the CDH field moving forward. The first is to conduct quantitative research to document the associations between corporate activities and changes in specific health outcomes. This could involve using causal diagrams to map out the expected associations between an activity (e.g., corporate influence in nutrition education) and an outcome (e.g., obesity) including all potential mediators (e.g., nutrition knowledge, eating behaviour), moderators (e.g., exposure to other sources of information) and confounders (e.g., fast-food permeation). Innovative methods such as difference-in-differences (DiD) [74] designs may be used to mitigate some of the challenges associated with determining statistical associations within causal pathways that involve multiple mediators, moderators, and confounders. In this context, DiD could be used to evaluate changes in health outcomes in a population in which restrictions are implemented on a corporate activity of interest compared to an analogous population in which the activity is not restricted, affording identification of the effect of the restriction (and thus, the potential impact of the activity) [75].

The second research priority is to use the CDH concept to advance research on contemporary and emerging industries whose products pose a potential threat to health, such as the social media, cannabis, gambling, and e-cigarette industries. We recommend collaboration between CDH scholars (conceptual experts) and public health experts already studying these industries from different perspectives (content experts) to advance this priority. Investigative journalists are also potential collaborators given that they often conduct detailed investigations of these industries.

The third priority is to increase efforts to document corporate activities influencing health in LMICs, where health harms related to the CDH are most likely to be concentrated. In addition to working directly with scholars and advocates based in LMICs and increasing capacity in these regions, this may involve advocating for more transparent data from corporations on the activities they are conducting in these countries. For example, CDH scholars could work with the *Global Reporting Initiative*, an organization that develops ESG standards that thousands of companies use to report data on their environmental and social impacts [76], to support the development of health-related corporate ESG disclosures that reflect the CDH. Given that some ESG disclosures have become legally mandated in certain regions (i.e., the European Union) [77], getting health on the ESG agenda has the potential to open up useful and comprehensive data sources for future CDH research. However, to ensure this data is relevant to LMICs, emphasis should be placed on corporate disclosure of health-relevant activities across the entire supply chain (as opposed to, for example, focusing on activities in the location where the company is headquartered). These efforts, alongside ongoing projects such as INFORMAS (a global effort to monitor public and private sector activities related to food environments [78]) may assist us in better understanding the ways in which the CDH manifest in LMICs.

Overall, addressing these three research priorities will be important to enhancing the comprehensiveness and usefulness of the CDH concept as a lens to understanding the root causes of population health and health equity.

## Supporting information

S1 File (DOCX)
