# Supplementary material for: Characteristics of commercial determinants of health research on corporate activities: A scoping review
Source: PLoS One. 2024 Apr 26;19(4):e0300699. doi: 10.1371/journal.pone.0300699 (PMC11051660; doi:10.1371/journal.pone.0300699)
Supplement: S1 File — (DOCX) [file pone.0300699.s001.docx]

**Supporting Information**

Table of Contents

[Appendix 1: Search Strategy Used in Review 1](#_Toc163135307)

[Appendix 2: Rules Used to Guide Classification of Articles by Methods, Industries, and Regions 2](#_Toc163135308)

[Appendix 3: Table Describing the Characteristics of Included Articles 4](#_Toc163135309)

[Appendix 4: Overlap Between Literature Included in this Review and Literature Included in de Lacy-Vawdon and Livingstone’s Review 16](#_Toc163135310)

[Appendix 5. PRISMA Checklist 18](#_Toc163135311)

# **Appendix 1:** Search Strategy Used in Review

**Databases:** Scopus, OVID Medline, Ovid Embase, Ovid Global Health

Searched from inception on Sept 13, 2022

| Database | Search String |
| --- | --- |
| Scopus | TITLE-ABS-KEY ((( corporat* OR commercial*) AND determinant* ) OR "corporate political activity" OR "corporate political activities") AND ( health* OR disease* OR wellbeing OR "well being" OR morbidit* OR mortalit* OR "life expectanc* " OR DALY OR DALYs OR "disability-adjusted life year" OR "disability-adjusted life years" OR QALY OR QALYs OR "quality-adjusted life year" OR "quality-adjusted life years") |
| OVID Medline(R) | ((( corporat* OR commercial*) AND determinant*) OR "corporate political activity" OR "corporate political activities") AND ( health* OR disease* OR wellbeing OR "well being" OR morbidit* OR mortalit* OR "life expectanc*" OR DALY OR DALYs OR "disability-adjusted life year" OR "disability-adjusted life years" OR QALY OR QALYs OR "quality-adjusted life year" OR "quality-adjusted life years").mp. [mp=title, abstract, original title, name of substance word, subject heading word, floating sub-heading word, keyword heading word, organism supplementary concept word, protocol supplementary concept word, rare disease supplementary concept word, unique identifier, synonyms]. |
| Embase | (((corporat* OR commercial*) AND determinant*) OR "corporate political activity" OR "corporate political activities") AND ( health* OR disease* OR wellbeing OR "well being" OR morbidit* OR mortalit* OR "life expectanc*" OR DALY OR DALYs OR "disability-adjusted life year" OR "disability-adjusted life years" OR QALY OR QALYs OR "quality-adjusted life year" OR "quality-adjusted life years").mp. [mp=title, abstract, heading word, drug trade name, original title, device manufacturer, drug manufacturer, device trade name, keyword heading word, floating subheading word, candidate term word] |
| Global Health | ((( corporat* OR commercial*) AND determinant*) OR "corporate political activity" OR "corporate political activities") AND ( health* OR disease* OR wellbeing OR "well being" OR morbidit* OR mortalit* OR "life expectanc*" OR DALY OR DALYs OR "disability-adjusted life year" OR "disability-adjusted life years" OR QALY OR QALYs OR "quality-adjusted life year" OR "quality-adjusted life years").mp. [mp=abstract, title, original title, broad terms, heading words, identifiers, cabicodes] |

# **Appendix 2:** Rules Used to Guide Classification of Articles by Methods, Industries, and Regions

***Method Classifications:***

| Method Classification^a,b^ | Description |
| --- | --- |
| Conceptual | Articles that did not use empirical methodology and instead engaged in processes such as describing the CDH, proposing new frameworks, or using previous frameworks to illuminate the CDH in new industries. |
| Commentaries | Articles that identified themselves as commentaries or editorials. |
| Responses | Articles that identified themselves as responses to other articles. |
| Reviews | Articles that reported review methodology and described their literature search and selection process. |
| Qualitative | Articles reporting the findings from qualitative analysis of data sources such as documents and interviews. |
| Quantitative | Articles that reported the findings of quantitative analysis. |
| Quantitative Protocol | Articles that proposed a study design for a quantitative study. |
| Mixed Methods | Articles that reported using both qualitative and quantitative methods. |
| Scale Development | Articles reporting on the development of a scale. |
| Scale Application | Articles that involved the application of a previously developed scale. |
| Workshop | Articles reporting on the findings from a scientific workshop. |

^a^For simplicity, articles were not categorized into multiple categories and were instead classified into the most fitting category. For example, a review study that engaged in qualitative analysis of the included documents was classified as a review and not a qualitative study.

^b^Some articles reported using case study methodology. These articles were classified as either conceptual if they did not report any empirical methodology or by their respective methods (e.g., qualitative) if they reported using empirical methodology.

***Industry Classifications:***

Articles were classified to a particular industry if they dedicated a significant portion of the article to discussing that specific industry or a relevant sub-industry (e.g., a study on the meat industry was classified to ‘food and beverage’). If the article discussed multiple industries in this way, they were classified to each respective industry. Articles were classified as ‘general’ if they reported on corporate activities more broadly (sometimes, these articles used certain industries as illustrative examples).

***Region Classifications:***

Articles were classified to a particular region if they studied corporate activities occurring in that region, collected data from sources or participants from that region, or otherwise focused their article on that particular region. Articles were classified as ‘global’ if they specifically reported a global focus and ‘not applicable’ if they did not report a global or regional focus (for e.g., conceptual articles that described corporate activities generally).

***Funding Status:***

An article was counted as having received funding if the authors reported funding that was specifically received for the research described in the respective article. If the authors reported funding for the Investigators (i.e., salary-support) that was not specific to the research in question, this was not counted as having received funding. In cases where it was unclear whether the funding was specific to the research described in the respective article, the first author (R.B.) followed up with the authors of the respective article to clarify the funding status. In cases where there was no section to report funding in the respective article, this was counted as having not declared funding.

***Reported Funding Sources Classifications:***

Reported funding sources were classified according to whether the funder was an educational institution (e.g., University College London); a government entity (e.g., São Paulo Research Foundation); a philanthropic organization (referring to philanthropic organizations, non-profit organizations, and charities) (e.g., U.S. Right to Know); or an international governance organization (e.g., The World Health Organization). No for-profit funders were reported in the included articles.

***Conflicts of Interest (COI):***

An article was counted as having reported a conflict of interest if the authors reported anything within the COI sections of the relevant articles. An article was counted as not having reported a COI if the authors specifically reported that there were no COIs to declare or if there was no section to report COIs with the respective article (i.e., not reported).

***Open-Access Status:***

Whether or not the article was published open-access was determined by using *The Lens* scholarly database (https://www.lens.org/), which classifies articles by their open-access status. For articles in which this information was not available on *Lens*, the first author (R.B.) checked the original article for its open-access status (i.e., looked for the presence of a Creative Commons license and ability to access without institutional login).

# **Appendix 3**: Table Describing the Characteristics of Included Articles

| Author(s) (Year) | Title | Type of Article | Industry/ies | Regions | Research-Specific Funding Reported? (Y/N) | Type of Funder | Reported Conflict of Interest? (Y/N)^a^ | Open-Access?  (Y/N) |
| --- | --- | --- | --- | --- | --- | --- | --- | --- |
| Millar, J. (2013) | The Corporate Determinants of Health: How Big Business Affects Our Health, and the Need for Government Action! | Commentary | General, with focus on food and beverage | Canada | N | - | N | Y |
| Kickbusch, I. Allen, L., Franz, C. (2016) | The commercial determinants of health | Commentary | General | - | N | - | N | Y |
| Buse, K., Tanaka, S., Hawkes, S (2017) | ﻿Healthy people and healthy profits? Elaborating a conceptual framework for governing the commercial determinants of non-communicable diseases and identifying options for reducing risk exposure | Conceptual | Tobacco, ultra-processed foods, alcohol | Global | Y | Educational Institution | N | Y |
| Knai, C. et al. (2018) | Systems Thinking as a Framework for Analyzing Commercial Determinants of Health | Conceptual | General | - | Y | Philanthropic | N | Y |
| McKee, M, Stuckler, D. (2018) | Revisiting the corporate and commercial determinants of health | Conceptual | General | - | N | - | N | Y |
| Wiist, W. (2019) | Mechanisms Underlying Corporations as Determinants of Health | Response | General, focus on tobacco | - | N | - | Y | Y |
| Brown, T. (2019) | Legislative capture: A critical consideration in the commercial determinants of public health | Conceptual | General, with focus on alcohol | Australia | N | - | N | N |
| Ireland, R., Chambers, S., Bunn, C. (2019) | Exploring the relationship between Big Food corporations and professional sports clubs: a scoping review | Review | Food and beverage | Global | Y | Educational Institution | N | Y |
| Toebes, B.,  Patterson, D. (2019) | Human rights and Non-Communicable Diseases: Controlling Tobacco and Promoting Healthy Diets (Book Chapter) | Conceptual | General, focus on tobacco and food and beverage | Global | N | - | N | N |
| Hessari, N. et al. (2019) | Recruiting the “heavy-using loyalists of tomorrow”: An analysis of the aims, effects and mechanisms of alcohol advertising, based on advertising industry evaluations | Qualitative | Alcohol | United Kingdom | Y | Educational Institution | N | Y |
| Peres, M et al. (2019) | Oral diseases: a global public health challenge | Conceptual | Food and beverage | Global | N | - | N | Y |
| Battams, S. & Townsend, B. (2019) | Power asymmetries, policy incoherence and noncommunicable disease control - a qualitative study of policy actor views | Qualitative | General, focus on tobacco, alcohol, food and beverage | Global, Switzerland, Australia, Malaysia | Y | Educational Institution | N | N |
| Madureira Lima, J. & Galea, S. (2019) | The Corporate Permeation Index – A tool to study the macrosocial determinants of Non-Communicable Disease | Scale Development | General | Global | N | - | N | Y |
| Hessari, N. et al. (2019) | Public Meets Private: Conversations Between Coca-Cola and the CDC | Qualitative | Beverage | United States | Y | Philanthropic | Y | Y |
| Ireland, R. et al. (2019) | Commercial determinants of health: Advertising of alcohol and unhealthy foods during sporting events | Conceptual | Alcohol, food and beverage | United Kingdom | Y | Government Entity | Y | Y |
| Kadandale, S., Marten, R., Smith, R. (2019) | The palm oil industry and noncommunicable diseases | Conceptual | Food (palm oil) | Global | N | - | N | Y |
| Kasture, A et al. (2019) | Benchmarking the commitments related to population nutrition and obesity prevention of major food companies in New Zealand | Scale Application | Food and beverage | New Zealand | N | - | N | Y |
| Fooks, G. et al. (2019) | Corporations' use and misuse of evidence to influence health policy: A case study of sugar-sweetened beverage taxation | Qualitative | Beverage | South Africa | N | - | N | Y |
| Kenworthy, N. (2019) | Crowdfunding and global health disparities: an exploratory conceptual and empirical analysis | Conceptual | Crowdfunding | Global | Y | Educational Institution | N | Y |
| Rochford, C., Tenneti, N., Moodie, R. (2019) | Reframing the impact of business on health: the interface of corporate, commercial, political and social determinants of health | Commentary | General | - | N | - | N | Y |
| Mialon et al. (2020) | ‘We must have a sufficient level of profitability’: food industry submissions to the French parliamentary inquiry on industrial food | Qualitative | Food & beverage | France | Y | Government entity | N | N |
| Eastmure, E., Cummins, S., Sparks, L. (2020) | Non-market strategy as a framework for exploring commercial involvement in health policy: A primer | Conceptual | General |  | N |  | N | Y |
| Maani, N., Abdalla, S., Galea, S. (2020) | The firearm industry as a commercial determinant of health | Commentary | Firearm | United States | N | - | N | N |
| Lacy-Nicholas, J., Scrinis, G., Carey, R. (2020) | The politics of voluntary self-regulation: Insights from the development and promotion of the Australian Beverages Council's Commitment | Qualitative | Beverage | Australia | N | - | N | Y |
| Brown, T (2020) | Public health vs alcohol industry compliance laws: A case of regulatory capture? | Conceptual | Alcohol | Australia | N | - | N | N |
| Sacks, G et al. (2020) | Benchmarking the nutrition-related policies and commitments of major food companies in Australia, 2018 | Scale Application | Food and beverage | Australia | N | - | Y | Y |
| Hastings et al. (2020) | Selling second best: how infant formula marketing works | Qualitative | Baby food | United Kingdom, Continental Europe, North America, Australia and New Zealand | Y | International Governance Organization | N | Y |
| De Lacy-Vawdon, C. & Livingstone, C. (2020) | Defining the commercial determinants of health: a systematic review | Review | General | - | N | - | N | Y |
| Mialon, M., Crosbie, E., Sacks, G. (2020) | Mapping of food industry strategies to influence public health policy, research and practice in South Africa | Qualitative | Food and beverage | South Africa | Y | Philanthropic | N | Y |
| Mialon, M. et al. (2020) | Arguments used by trade associations during the early development of a new front-of-pack nutrition labelling system in Brazil | Qualitative | Food and beverage | Brazil | Y | Government Entity | N | N |
| Hill, S., Friel, S. (2020) | ‘As Long as It Comes off as a Cigarette Ad, Not a Civil Rights Message’: Gender, Inequality and the Commercial Determinants of Health | Conceptual | Tobacco, alcohol |  | N |  | N | Y |
| Mialon, M. (2020) | An overview of the commercial determinants of health | Review | General | - | N | - | N | Y |
| Tanrikulu, H. et al. (2020) | Corporate political activity of the baby food industry: The example of Nestlé in the United States of America | Qualitative | Baby food | United States | N | - | N | Y |
| Hoe, C. et al. (2020) | Drink, but don't drive? The alcohol industry's involvement in global road safety | Qualitative | Alcohol | Global | Y | International Governance Organization | N | Y |
| Mialon, M. (2020) | Food industry political practices in Chile: “the economy has always been the main concern” | Qualitative | Food and beverage | Chile | Y | Educational Institution; Government Entity | N | Y |
| Lauber et al., (2020) | Non-communicable disease governance in the era of the sustainable development goals: A qualitative analysis of food industry framing in WHO consultations | Qualitative | Food and beverage | Global | N | - | N | Y |
| Jamieson, L., Gibson, B., Thomson, W. (2020) | Oral health inequalities and the corporate determinants of health: A commentary | Commentary | Food and beverage, alcohol, tobacco | - | N | - | N | Y |
| Karim, Kruger & Hofman (2020) | Industry strategies in the parliamentary process of adopting a sugar-sweetened beverage tax in South Africa: a systematic mapping | Qualitative | Beverage | South Africa | Y | Government entity | N | Y |
| Mialon et al. (2020) | “The architecture of the state was transformed in favour of the interests of companies”: corporate political activity of the food industry in Colombia | Qualitative | Food and beverage | Colombia | Y | Educational Institution; Government Entity | N | Y |
| Petticrew et al. (2020) | Dark Nudges and Sludge in Big Alcohol: Behavioral Economics, Cognitive Biases, and Alcohol Industry Corporate Social Responsibility | Qualitative | Alcohol | - | N | - | N | Y |
| McCarthy et al. (2020) | ‘It’s a tradition to go down to the pokies on your 18th birthday’ – the normalisation of gambling for young women in Australia | Qualitative | Gambling | Australia | Y | Government Entity | N | Y |
| Howse, E et al. (2021) | Air pollution and the noncommunicable disease prevention agenda: Opportunities for public health and environmental science | Commentary | General | - | N | - | N | Y |
| van Schalkwyk, M et al. (2021) | A public health approach to gambling regulation: countering powerful influences | Commentary | Gambling | United Kingdom | N | - | Y | Y |
| Cossez, E., Baker, P., Mialon, M. (2021) | ‘The second mother’: How the baby food industry captures science, health professions and civil society in France | Qualitative | Baby food | France | N | - | N | Y |
| Madden, M. & McCambridge, J. (2021) | Alcohol marketing versus public health: David and Goliath? | Commentary | Alcohol | Global | N | - | N | Y |
| Fisher, L et al. (2021) | Barriers and opportunities to restricting marketing of unhealthy foods and beverages to children in Nepal: a policy analysis | Qualitative | Food and beverage | Nepal | Y | Educational Institution; Government Entity | N | Y |
| Stubbs, T. (2021) | Commercial determinants of youth smoking in ASEAN countries: A narrative review of research investigating the influence of tobacco advertising, promotion, and sponsorship | Review | Tobacco | ASEAN (Association of Southeast Asian Nations) | Y | Educational Institution | N | Y |
| Mialon, M et al. (2021) | Beyond nutrition and physical activity: food industry shaping of the very principles of scientific integrity | Qualitative | General, food and beverage | - | Y | Philanthropic | N | Y |
| Wood, B., Baker, P., Sacks, G. (2021) | Conceptualising the Commercial Determinants of Health Using a Power Lens: A Review and Synthesis of Existing Frameworks | Review | General | - | N | - | N | Y |
| Chavez-Ugalde, Y et al. (2021) | Conceptualizing the commercial determinants of dietary behaviors associated with obesity: A systematic review using principles from critical interpretative synthesis | Review | Food and beverage | - | N | - | N | Y |
| Lauber, K et al. (2021) | Corporate political activity in the context of unhealthy food advertising restrictions across Transport for London: A qualitative case study | Qualitative | Food and beverage | United Kingdom | N | - | Y | Y |
| Freudenberg, N et al. (2021) | Defining Priorities for Action and Research on the Commercial Determinants of Health: A Conceptual Review | Review | General | - | Y | Government entity | N | Y |
| Zenone, M et al. (2021) | How does the British Soft Drink Association respond to media research reporting on the health consequences of sugary drinks? | Qualitative | Beverage | United Kingdom | N | - | N | Y |
| Milaon, M et al. (2021) | Involvement of the food industry in nutrition conferences in Latin America and the Caribbean | Qualitative | Food and beverage | Latin America, Caribbean | N | - | Y | Y |
| Hunt, D. (2021) | How food companies use social media to influence policy debates: A framework of Australian ultra-processed food industry Twitter data | Qualitative | Food and beverage | Australia | N | - | N | Y |
| Wood, B et al. (2021) | Market strategies used by processed food manufacturers to increase and consolidate their power: a systematic review and document analysis | Review | Food and beverage | - | N | - | N | Y |
| Wood, B et al. (2021) | The double burden of maldistribution: a descriptive analysis of corporate wealth and income distribution in four unhealthy commodity industries | Quantitative | Food and beverage, Alcohol, Tobacco, Fossil Fuels | Global, USA | N | - | N | N |
| Lacy-Nichols, J. & Marten, R. (2021) | Power and the commercial determinants of health: Ideas for a research agenda | Commentary | General | - | N | - | N | Y |
| Zenone, M. & Kenworthy, N. (2021) | Pre-emption strategies to block taxes on sugar-sweetened beverages: A framing analysis of Facebook advertising in support of Washington state initiative-1634 | Qualitative | Beverage | United States | N | - | N | Y |
| Loewenson, R. (2021) | Rethinking the Paradigm and Practice of Occupational Health in a World Without Decent Work: A Perspective From East and Southern Africa | Commentary | General, Mining | East and Southern Africa | N | - | N | Y |
| Knai, C et al. (2021) | The case for developing a cohesive systems approach to research across unhealthy commodity industries | Workshop | General | - | Y | Philanthropic | N | Y |
| Mendly-Zambo, Z., Raphael, D., Taman, A. (2021) | Take the money and run: how food banks became complicit with Walmart Canada’s hunger producing employment practices | Conceptual | Retail | Canada | N | - | N | N |
| Hyder, A et al. (2021) | The COVID-19 Pandemic Exposes Another Commercial Determinant of Health: The Global Firearm Industry | Commentary | Firearm | - | Y | Educational Institution | N | Y |
| Campbell, N et al. (2021) | The Gift of Data: Industry-Led Food Reformulation and the Obesity Crisis in Europe | Qualitative | Food and beverage | United Kingdom, Portugal, Ireland, Germany, France | N | - | N | N |
| Zenone, M., Kenworthy, N., Barbic, S. (2021) | The Paradoxical Relationship Between Health Promotion and the Social Media Industry | Commentary | Social media | - | N | - | Y | Y |
| Gerritsen, S et al. (2021) | The Timing, Nature and Extent of Social Media Marketing by Unhealthy Food and Drinks Brands During the COVID-19 Pandemic in New Zealand | Qualitative | Food and beverage | New Zealand | Y | Philanthropic | N | Y |
| Klein, D. & Lima, J. (2021) | The Prison Industrial Complex as a Commercial Determinant of Health | Commentary | Prison | United States | N | - | N | Y |
| McHardy, J. (2021) | The WHO FCTC's lessons for addressing the commercial determinants of health | Commentary | General, tobacco | Global | N | - | Y | Y |
| Russ, K et al. (2021) | What You Don’t Know About the Codex Can Hurt You: How Trade Policy Trumps Global Health Governance in Infant and Young Child Nutrition | Mixed methods | Baby food | Global | Y | Philanthropic | Y | Y |
| Baker, P et al. (2021) | Breastfeeding, first-food systems and corporate power: a case study on the market and political practices of the transnational baby food industry and public health resistance in the Philippines | Qualitative | Baby food | Philippines | Y | International Governance Organization | Y | Y |
| Baker, P et al. (2021) | First-food systems transformations and the ultra-processing of infant and young child diets: The determinants, dynamics and consequences of the global rise in commercial milk formula consumption | Review | Baby food | Global | Y | International Governance Organization | N | Y |
| Baker, P et al. (2021) | Globalization, first-foods systems transformations and corporate power: a synthesis of literature and data on the market and political practices of the transnational baby food industry | Review | Baby food | Global | Y | International Governance Organization | Y | Y |
| Jones et al. (2021) | Disrupting the commercial determinants of health; Chapter 5 in “Australia in 2030 – What is our path to health for all?” (Supplement) | Conceptual | General | Australia | Y | Government Entity | N | Y |
| Maani et al. (2021) | The new WHO Foundation – global health deserves better | Commentary | General | Global | N | - | Y | Y |
| Maani et al. (2021) | The need for a conceptual understanding of the macro and meso commercial determinants of health inequalities | Commentary | General, Alcohol, Tobacco | - | N | - | N | Y |
| Brisbois et al. (2021) | Mining, colonial legacies, and neoliberalism: A political ecology of health knowledge | Conceptual | Extractive | Canada | N | - | Y | Y |
| Jia et al. (2021) | #SupportLocal: how online food delivery services leveraged the COVID-19 pandemic to promote food and beverages on Instagram | Mixed Methods | Food and beverages | Australia, New Zealand, United Kingdom, United States, Canada | N | - | N | Y |
| Mialon et al. (2021) | ‘I had never seen so many lobbyists’: food industry political practices during the development of a new nutrition front-of-pack labelling system in Colombia | Qualitative | Food and beverage | Colombia | Y | Educational Institution; Government Entity | N | Y |
| Dall’Alba & Rocha (2021) | Brazil’s response to COVID-19: commercial determinants of health and regional inequities matter | Commentary | General | Brazil | N | - | N | Y |
| Diderichsen et al. (2021) | Beyond ‘commercial determinants’: shining a light on privatization and political drivers of health inequalities | Commentary | General | Sweden, Denmark | N | - | N | Y |
| Yates et al. (2021) | Trust and responsibility in food systems transformation. Engaging with Big Food: marriage or mirage? | Conceptual | Food and beverage | Global | N | - | N | Y |
| Maani et al. (2021) | The Commercial Determinants of Three Contemporary National Crises: How Corporate Practices Intersect With the  COVID-19 Pandemic, Economic Downturn, and Racial Inequity | Commentary | General | United States | N | - | N | Y |
| Van Schalkwyk et al. (2021) | Our Postpandemic World: What Will It Take to Build a Better Future for People and Planet? | Commentary | General | Global | N | - | N | Y |
| Adams, Rychert, & Wilkins (2021) | Policy inﬂuence and the legalized cannabis industry: learnings from other addictive consumption industries | Conceptual | Cannabis | New Zealand | N | - | Y | N |
| Boatwright et al. (2021) | The Politics of Regulating Foods for Infants and Young Children: A Case Study on the Framing and Contestation of Codex Standard-Setting Processes on Breast-Milk Substitutes | Mixed Methods | Baby food | Global | Y | International Governance Organization | N | Y |
| Watts, Burton & Freman (2021) | ‘The last line of marketing’: Covert tobacco marketing tactics as revealed by former tobacco industry employees | Qualitative | Tobacco | Australia | Y | Philanthropic | N | Y |
| Gillespie et al. (2021) | Conceptualising changes to tobacco and alcohol policy as affecting a single interlinked system | Workshop | Tobacco, alcohol | United Kingdom | Y | Philanthropic; Government Entity; Educational Institution | N | Y |
| Jamieson et al. (2021) | Neoliberalism and Indigenous oral health inequalities: a global perspective | Commentary | Tobacco, food and beverage | Global | N | - | N | N |
| Hill et al. (2021) | From silos to policy coherence: tobacco control, unhealthy commodity industries and the commercial determinants of health | Commentary | Tobacco, General | - | N | - | Y | Y |
| McCarthy, S et al. (2022) | Electronic gambling machine harm in older women: a public health determinants perspective | Qualitative | Gambling | Australia | N | - | Y | N |
| Lee, K et al. (2022) | Measuring the Commercial Determinants of Health and Disease: A Proposed Framework | Scale Development | General | - | Y | Government Entity | N | Y |
| Allen, Wigley, and Homer (2022) | Assessing the association between Corporate Financial Influence and implementation of policies to tackle commercial determinants of non-communicable diseases: A cross-sectional analysis of 172 countries | Quantitative | General | Global | N | - | N | Y |
| Buse, Mialon, Jones (2022) | Thinking politically about UN political declarations: A recipe for healthier commitments – free of commercial interests | Conceptual | General | - | N | - | N | Y |
| Clare, Maani, and Milner (2022) | Meat, money and messaging: How the environmental and health harms of red and processed meat consumption are framed by the meat industry | Qualitative | Food (meat) | United Kingdom | N | - | N | N |
| Barlow & Allen (2022) | The impact of trade and investment agreements on the implementation noncommunicable disease policies, 2014-2019: protocol for a statistical study (preprint) | Quantitative Protocol | Tobacco, alcohol, food and beverage | Global | N | - | N | Y |
| DuPont-Reyes, Hernandez-Munoz, and Tang (2022) | TV advertising, corporate power, and Latino health disparities | Mixed Methods | Alcohol, tobacco, food and beverage, pharmaceutical | United States | N | - | N | N |
| Gokani et al. (2022) | UK Nutrition Research Partnership ‘Hot Topic’ workshop report: A ‘game changer’ for dietary health – addressing the implications of sport sponsorship by food businesses through an innovative interdisciplinary collaboration | Workshop | Food and beverage | United Kingdom | Y | Government Entity | N | Y |
| Hird et al. (2022) | Understanding the long-term policy influence strategies for the tobacco industry: two contemporary case studies | Conceptual | Tobacco | - | Y | Philanthropic | N | Y |
| Montiel et al. (2022) | Tracing the connections between international business and communicable diseases | Conceptual | General | Global | Y | Educational Institution | N | Y |
| Fooks & Godziewski (2022) | The World Health Organization, Corporate Power, and the Prevention and Management of Conflicts of Interest in Nutrition Policy: Comment on “Towards Preventing and Managing Conflict of Interest in Nutrition Policy? An Analysis of Submissions to a Consultation on a Draft WHO Tool” | Commentary | Food and beverage | Global | N | - | N | Y |
| Zenone, Kenworthy, & Maani (2022) | The Social Media Industry as a Commercial Determinant of Health | Commentary | Social media |  | N | - | N | Y |
| Mialon et al. (2022) | Conflicts of interest for members of the U.S. 2020 Dietary Guidelines Advisory Committee | Quantitative | Food and beverage | United States | Y | Philanthropic | N | Y |
| Passini et al. (2022) | Conflict of interests in the scientific production of Vitamin D and COVID-19: A Scoping Review | Review | Food, diagnostics, pharmaceutical | - | Y | Educational Institution | N | Y |
| Ramsbottom et al. (2022) | Food as harm reduction during a drinking session: reducing the harm or normalising harmful use of alcohol? A qualitative comparative analysis of alcohol industry and non-alcohol industry-funded guidance | Qualitative | Alcohol | - | N | - | N | Y |
| Rose, Reeve & Charlton (2022) | Barriers and Enablers for Healthy Food Systems and Environments: The Role of Local Governments | Review | Food and beverage | - | N | - | N | Y |
| Steele et al. (2022) | Confronting potential food industry ‘front groups’: case study of the international food information Council’s nutrition communications using the UCSF food industry documents archive | Qualitative | Food and beverage | Global | Y | Philanthropic | Y | Y |
| De Lacy-Vawdon, Vandenberg, & Livingstone (2022) | Recognising the elephant in the room: the commercial determinants of health | Commentary | General | - | N | - | N | Y |
| Wiist, W. (2022) | The Foundations of Corporate Strategies: Comment on “‘Part of the Solution’: Food Corporation Strategies for Regulatory Capture and Legitimacy” | Commentary | General | United States | N | - | Y | Y |
| Maani et al. (2022) | Manufacturing doubt: Assessing the effects of independent vs industry-sponsored messaging about the harms of fossil fuels, smoking, alcohol, and sugar sweetened beverages | Quantitative | Alcohol, tobacco, fossil fuels, sugar-sweetened beverages | United Kingdom | Y | Government Entity | N | Y |
| Hoe et al. (2022) | Strategies to expand corporate autonomy by the tobacco, alcohol and sugar-sweetened beverage industry: a scoping review of reviews | Review | Tobacco, alcohol, sugar-sweetened beverages | - | Y | Philanthropic | N | Y |
| Freudenberg (2022) | Responding to Food Industry Initiatives to Be “Part of the Solution”  Comment on “‘Part of the Solution’: Food Corporation Strategies for Regulatory Capture and Legitimacy” | Response | Food and beverage | - | N | - | N | Y |
| Kroker-Lobos et al. (2022) | Two countries, similar practices: The political practices of the food industry influencing the adoption of key public health nutrition policies in Guatemala and Panama | Qualitative | Food | Guatemala, Panama | Y | Educational Institution; Government Entity | N | Y |
| Leimbigler et al. (2022) | Social, political, commercial, and corporate determinants of rural health equity in Canada: an integrated framework | Commentary | General | Canada | Y | Educational Institution | N | Y |
| Liber (2022) | Using Regulatory Stances to See All the Commercial Determinants of Health | Conceptual | General, Pharma/Diagnostics, Food & beverage, Housing, Tobacco (E-Cigarettes) | - | N | - | Y | Y |
| Wakefield, Glantz & Appollonio (2022) | Content Analysis of the Corporate Social Responsibility Practices of 9 Major Cannabis Companies in Canada and the US | Qualitative | Cannabis | United States, Canada | Y | Government Entity | N | Y |
| Van Schalkwyk, Hawkins & Pettigrew (2022) | The politics and fantasy of the gambling education discourse: An analysis of gambling industry-funded youth education programmes in the United Kingdom | Qualitative | Gambling | United Kingdom | Y | Government Entity | N | Y |

^a^Articles labelled with ‘N’ in this column either specifically declared that there was no related funding, reported funding that was not specific to the research (e.g., general support for investigators) or information on funding was not available (i.e., not reported). ‘Y’ indicated funding specific to the article in question was reported.

^b^Articles labelled with ‘N’ in this column either specifically declared no competing interests or information on competing interests was not available (i.e., not declared). ‘Y’ indicates that a competing interest was declared.

# **Appendix 4:** Overlap Between Literature Included in this Review and Literature Included in de Lacy-Vawdon and Livingstone’s Review

| Literature in de Lacy Vawdon and Livingstone (2020) Review^a^ | Included In This Review?^b^ |
| --- | --- |
| Buse, K., & Hawkes, S. (2015). Health in the sustainable development goals: ready for a paradigm shift?. *Globalization and health*, *11*(1), 1-8. |  |
| Buse, K., Tanaka, S., & Hawkes, S. (2017). Healthy people and healthy profits? Elaborating a conceptual framework for governing the commercial determinants of non-communicable diseases and identifying options for reducing risk exposure. *Globalization and health*, *13*(1), 1-12. |  |
| Collins, T., Mikkelsen, B., & Axelrod, S. (2019). Interact, engage or partner? Working with the private sector for the prevention and control of noncommunicable diseases. *Cardiovascular diagnosis and therapy*, *9*(2), 158. |  |
| Franz, C., & Kickbusch, I. (2018). The capital-NCD-nexus: the commercial determinants of health and global capital flows. *Eurohealth*, *24*(3), 21-25. |  |
| Freudenberg, N., & Galea, S. (2008). The impact of corporate practices on health: implications for health policy. *Journal of public health policy*, *29*, 86-104. |  |
| Freudenberg & Galea (2007). Corporate Practices (In Macrosocial Determinants of Population Health). *Springer*. |  |
| Hastings, G. (2015). Public health and the value of disobedience. *Public health*, *129*(8), 1046-1054. |  |
| Hastings, G. (2012). Why corporate power is a public health priority. *Bmj*, *345*. |  |
| IFMSA Policy Non-Communicable Diseases |  |
| Ireland, R., Bunn, C., Reith, G., Philpott, M., Capewell, S., Boyland, E., & Chambers, S. (2019). Commercial determinants of health: advertising of alcohol and unhealthy foods during sporting events. *Bulletin of the World Health Organization*, *97*(4), 290. |  |
| Kadandale, S., Marten, R., & Smith, R. (2019). The palm oil industry and noncommunicable diseases. *Bulletin of the World Health Organization*, *97*(2), 118. |  |
| Kickbusch, I. (2015). Addressing the commercial determinants is critical to emerging economies/Na area de saude, a abordagem dos fatores determinantes, de natureza comercial, e de importancia fundamental para os paises emergentes. *Ciência & Saúde Coletiva*, *20*(4), 968-970. |  |
| Kickbusch, I. (2013). A game change in global health: the best is yet to come. *Public Health Reviews*, *35*, 1-20. |  |
| Kickbusch, I. (2012). Addressing the interface of the political and commercial determinants of health. *Health Promotion International*, *27*(4), 427-428. |  |
| Kickbusch, I., Allen, L., & Franz, C. (2016). The commercial determinants of health. *The Lancet Global Health*, *4*(12), e895-e896. |  |
| Kickbusch, I., & Szabo, M. M. C. (2014). A new governance space for health. *Global Health Action*, *7*(1), 23507. |  |
| Knai, C., Petticrew, M., Mays, N., Capewell, S., Cassidy, R., Cummins, S., ... & Weishaar, H. (2018). Systems thinking as a framework for analyzing commercial determinants of health. *The Milbank Quarterly*, *96*(3), 472-498. |  |
| Kosinska & Ostlin. (2016). Building systematic approaches to intersectoral action in the WHO European Region. *Public Health Panorama* (Magazine editorial) |  |
| Madureira Lima, J., & Galea, S. (2018). Corporate practices and health: a framework and mechanisms. *Globalization and Health*, *14*(1), 1-12. |  |
| McKee, M., & Stuckler, D. (2018). Revisiting the corporate and commercial determinants of health. *American journal of public health*, *108*(9), 1167-1170. |  |
| Millar, J. S. (2013). The corporate determinants of health: how big business affects our health, and the need for government action!. *Canadian Journal of Public Health*, *104*, e327-e329. |  |
| Public Health Association of Australia. (2018). What are the determinants of health? [Web article] |  |
| Smith, J., Buse, K., & Gordon, C. (2016). Civil society: the catalyst for ensuring health in the age of sustainable development. *Globalization and Health*, *12*, 1-6. |  |
| Smith, K., Dorfman, L., Freudenberg, N., Hawkins, B., Hilton, S., Razum, O., & Weishaar, H. (2016). Tobacco, alcohol, and processed food industries–why do public health practitioners view them so differently?. *Frontiers in public health*, *4*, 64. |  |
| Sula-Raxhimi, E., Butzbach, C., & Brousselle, A. (2019). Planetary health: countering commercial and corporate power. *The Lancet Planetary Health*, *3*(1), e12-e13. |  |
| Thorn, M. (2018). Addressing power and politics through action on the commercial determinants of health. *Health Promotion Journal of Australia: Official Journal of Australian Association of Health Promotion Professionals*, *29*(3), 225-227. |  |
| Thurley (2017). Explaining the links between Commercial Determinants of Health and Chronic Diseases. European Public Health Alliance [Web editorial] |  |
| United Nations Department of Economic and Social Affairs, and United Nations Industrial Development Organization (2016). Report of the expert meeting in preparation for HLPF 2017 on readying institutions and policies for integrated approaches to implementation of the 2030 agenda. [Meeting report]. |  |
| West, R., & Marteau, T. (2013). Commentary on Casswell (2013): the commercial determinants of health. *Addiction (Abingdon, England)*, *108*(4), 686-687. |  |
| Wiist, W. H. (2006). Public health and the anticorporate movement: rationale and recommendations. *American Journal of Public Health*, *96*(8), 1370-1375. |  |
| World Health Organization (2017). 2. Convening to overcome commercial determinants of health (In Report of the Regional Director: The work of WHO in the Western Pacific Region 1 July 2016–30 June 2017) [Report section]. |  |
| World Health Organization Regional Office for Europe. (2016). Good governance for the health and wellbeing of all children and adolescents. [Conference paper] |  |

^a^de Lacy-Vawdon, C., & Livingstone, C. (2020). Defining the commercial determinants of health: a systematic review. *BMC Public Health*, *20*(1), 1-16. The authors employed a similar search strategy though with different inclusion criteria to our review.

^b^A black dot indicates the respective article was included in both reviews.

# **Appendix 5.** PRISMA Checklist

**Preferred Reporting Items for Systematic reviews and Meta-Analyses extension for Scoping Reviews (PRISMA-ScR) Checklist**

| **SECTION** | **ITEM** | **PRISMA-ScR CHECKLIST ITEM** | **REPORTED ON PAGE #** |
| --- | --- | --- | --- |
| **TITLE** | | | |
| Title | 1 | Identify the report as a scoping review. | Yes, title and methods |
| **ABSTRACT** | | | |
| Structured summary | 2 | Provide a structured summary that includes (as applicable): background, objectives, eligibility criteria, sources of evidence, charting methods, results, and conclusions that relate to the review questions and objectives. | Yes, in abstract |
| **INTRODUCTION** | | | |
| Rationale | 3 | Describe the rationale for the review in the context of what is already known. Explain why the review questions/objectives lend themselves to a scoping review approach. | Yes, introduction and beginning of methods |
| Objectives | 4 | Provide an explicit statement of the questions and objectives being addressed with reference to their key elements (e.g., population or participants, concepts, and context) or other relevant key elements used to conceptualize the review questions and/or objectives. | Yes, at the end of introduction |
| **METHODS** | | | |
| Protocol and registration | 5 | Indicate whether a review protocol exists; state if and where it can be accessed (e.g., a Web address); and if available, provide registration information, including the registration number. | Yes, methods |
| Eligibility criteria | 6 | Specify characteristics of the sources of evidence used as eligibility criteria (e.g., years considered, language, and publication status), and provide a rationale. | Yes, methods |
| Information sources* | 7 | Describe all information sources in the search (e.g., databases with dates of coverage and contact with authors to identify additional sources), as well as the date the most recent search was executed. | Yes, methods |
| Search | 8 | Present the full electronic search strategy for at least 1 database, including any limits used, such that it could be repeated. | Yes, Supporting Information |
| Selection of sources of evidence† | 9 | State the process for selecting sources of evidence (i.e., screening and eligibility) included in the scoping review. | Yes, methods |
| Data charting process‡ | 10 | Describe the methods of charting data from the included sources of evidence (e.g., calibrated forms or forms that have been tested by the team before their use, and whether data charting was done independently or in duplicate) and any processes for obtaining and confirming data from investigators. | Yes, methods |
| Data items | 11 | List and define all variables for which data were sought and any assumptions and simplifications made. | Yes, methods |
| Critical appraisal of individual sources of evidence§ | 12 | If done, provide a rationale for conducting a critical appraisal of included sources of evidence; describe the methods used and how this information was used in any data synthesis (if appropriate). | No quality assessment conducted, described in Methods |
| Synthesis of results | 13 | Describe the methods of handling and summarizing the data that were charted. | Yes, methods (data synthesis and analysis) |
| **RESULTS** | | | |
| Selection of sources of evidence | 14 | Give numbers of sources of evidence screened, assessed for eligibility, and included in the review, with reasons for exclusions at each stage, ideally using a flow diagram. | Yes, Figure 1 |
| Characteristics of sources of evidence | 15 | For each source of evidence, present characteristics for which data were charted and provide the citations. | Yes, Supporting Info |
| Critical appraisal within sources of evidence | 16 | If done, present data on critical appraisal of included sources of evidence (see item 12). | Not Applicable |
| Results of individual sources of evidence | 17 | For each included source of evidence, present the relevant data that were charted that relate to the review questions and objectives. | Yes, Supporting Info |
| Synthesis of results | 18 | Summarize and/or present the charting results as they relate to the review questions and objectives. | Yes, methods |
| **DISCUSSION** | | | |
| Summary of evidence | 19 | Summarize the main results (including an overview of concepts, themes, and types of evidence available), link to the review questions and objectives, and consider the relevance to key groups. | Yes, discussion |
| Limitations | 20 | Discuss the limitations of the scoping review process. | Yes, discussion |
| Conclusions | 21 | Provide a general interpretation of the results with respect to the review questions and objectives, as well as potential implications and/or next steps. | Yes, conclusion and recommendations |
| **FUNDING** | | | |
| Funding | 22 | Describe sources of funding for the included sources of evidence, as well as sources of funding for the scoping review. Describe the role of the funders of the scoping review. | Yes, Supporting Info and manuscript |

JBI = Joanna Briggs Institute; PRISMA-ScR = Preferred Reporting Items for Systematic reviews and Meta-Analyses extension for Scoping Reviews.

* Where *sources of evidence* (see second footnote) are compiled from, such as bibliographic databases, social media platforms, and Web sites.

† A more inclusive/heterogeneous term used to account for the different types of evidence or data sources (e.g., quantitative and/or qualitative research, expert opinion, and policy documents) that may be eligible in a scoping review as opposed to only studies. This is not to be confused with *information sources* (see first footnote).

‡ The frameworks by Arksey and O’Malley (6) and Levac and colleagues (7) and the JBI guidance (4, 5) refer to the process of data extraction in a scoping review as data charting*.*

§ The process of systematically examining research evidence to assess its validity, results, and relevance before using it to inform a decision. This term is used for items 12 and 19 instead of "risk of bias" (which is more applicable to systematic reviews of interventions) to include and acknowledge the various sources of evidence that may be used in a scoping review (e.g., quantitative and/or qualitative research, expert opinion, and policy document).

*From:* Tricco AC, Lillie E, Zarin W, O'Brien KK, Colquhoun H, Levac D, et al. PRISMA Extension for Scoping Reviews (PRISMAScR): Checklist and Explanation. Ann Intern Med. 2018;169:467–473. [doi: 10.7326/M18-0850](http://annals.org/aim/fullarticle/2700389/prisma-extension-scoping-reviews-prisma-scr-checklist-explanation).

*Downloaded from:* St. Michaels Knowledge Translation Program (<http://www.prisma-statement.org/documents/PRISMA-ScR-Fillable-Checklist_11Sept2019.pdf>).
